# Galectin-3 leads to attenuation of apoptosis through Bax heterodimerization in human thyroid carcinoma cells

**DOI:** 10.18632/oncotarget.2486

**Published:** 2014-09-16

**Authors:** Yosuke Harazono, Dhong Hyo Kho, Vitaly Balan, Kosei Nakajima, Tianpeng Zhang, Victor Hogan, Avraham Raz

**Affiliations:** ^1^ Departments of Oncology and Pathology, School of Medicine, Wayne State University, and Karmanos Cancer Institute, Detroit, MI; ^2^ Everon Biosciences, Buffalo, NY

**Keywords:** Galectin-3 (Gal-3), Bax, doxorubicin (DXR), apoptosis, thyroid cancer

## Abstract

Cancer cells survive escaping normal apoptosis and the blocks in apoptosis that keep cancer cells alive are promising candidates for targeted therapy. Galectin-3 (Gal-3) is, a member of the lectin family, which is involved in cell growth, adhesion, proliferation and apoptosis. It remains elusive to understand the role of Gal-3 on apoptosis in thyroid carcinoma cells. Here, we report that Gal-3 heterodimerizes Bax, mediated by the carbohydrate recognition domain (CRD) of Gal-3, leading to anti-apoptotic characteristic. Gal-3/Bax interaction was suppressed by an antagonist of Gal-3, in which in turn cells became sensitive to apoptosis. The data presented here highlight that Gal-3 is involved in the anti-apoptosis of thyroid carcinoma cells. Thus, it suggests that targeting Gal-3 may lead to an improved therapeutic modality for thyroid cancer.

## INTRODUCTION

Thyroid cancer is the cause of ~90% of all endocrine malignancies, and papillary thyroid carcinoma (PTC) is the most common subtype of this disease [1]. Most patients with PTC have a favorable prognosis, but a subset of patients suffers from recurrent disease that is refractory to surgical resection, radioactive iodine ablation and chemotherapeutic drugs. The ultimate goal of cancer research is to learn how to make cancer cells selectively die.

Since a hallmark of cancer is the dysregulation of programmed cell death, cancer cells survive with the evasion of apoptosis. Also the resistance to apoptosis can cause resistance to conventional cytotoxic therapy [2]. Thus, cellular apoptotic pathways are attractive candidates for targeted therapies. The alteration in expression of B cell lymphoma-2 (Bcl-2) family protein members, which govern the commitment to programmed cell death, is one of the suggested mechanisms for resistance to apoptosis. The Bcl-2 family proteins harboring the Bcl-2 homology (BH) domains serve as pro- and anti-apoptotic executors [3]. Many subsequent mechanistic studies led to the current model that anti-apoptotic family members sequestered the BH3 domain of pro-apoptotic proteins (Bax/Bak) in the mitochondria, and relieved them in response to diverse intrinsic and/or extracellular stress [4]. Ultimately Bax/Bak is oligomerized, leading to mitochondrial outer membrane permeabilization (MOMP) in which cytochrome c is released into the cytoplasm with the subsequent release of apoptogenic factors that exert combined processing of DNA fragmentation [5, 6].

The galectins are a family of mammalian β-galactoside binding proteins that share highly conserved carbohydrate recognition domain (CRD). To date, 15 galectin members have been identified and are associated with dysregulation in cancer cell growth that include defective apoptosis and cell-cycle alterations in carcinogenesis [7, 8]. Galectin-3 (Gal-3) is the only member of the chimera-type galectin subgroup and contains a single CRD similar to other galectins as well as small N-terminal part and collagen-like sequence. Gal-3 is broadly expressed in many tumor cells and is a good diagnostic marker for differentiated thyroid cancer [9]. It is observed both inside and outside cells, and exerts multifunctional roles in transformation, survival and anti-apoptosis [10]. The mechanisms by which Gal-3 regulates these processes have been elucidated with cues of Gal-3 interacting proteins such as oncogenic Ras and activation of pro-survival signaling including phosphatidylinositol 3-kinase (PI3K) and mitogen activated protein kinases (MAPKs) pathways. The anti-apoptotic activity of Gal-3 has been shown to translocate either from the cytosol or the nucleus to the mitochondria in response to apoptotic stimuli. Supporting this, truncated protein and serine mutant of Gal-3 had increased sensitivity to apoptotic stimuli. With the respect to a binding motif, Gal-3 has unique aspartate-tryptophan-glycine-arginine (NWGR), shown as a conservative motif of the Bcl-2 family, and its substitution mutant loses apoptosis-resistant properties in overexpression studies [11, 12]. The NWGR motif in Bcl-2 is also significant for Bcl-2/Bax heterodimerization, which prevents Bax oligomerization [13]. However, how this motif of Gal-3 is conducive to apoptotic resistance is still unclear.

In this study we examined the anti-apoptotic role of Gal-3 in thyroid carcinoma cells and demonstrate that Gal-3 interacts with pro-apoptotic Bax of the canonical apoptotic pathway, leading to inhibition of apoptosis.

## RESULTS

### Gal-3 expression contributes to cell growth and cell death in thyroid carcinoma cells in response to DXR treatment

**Fig.1:**
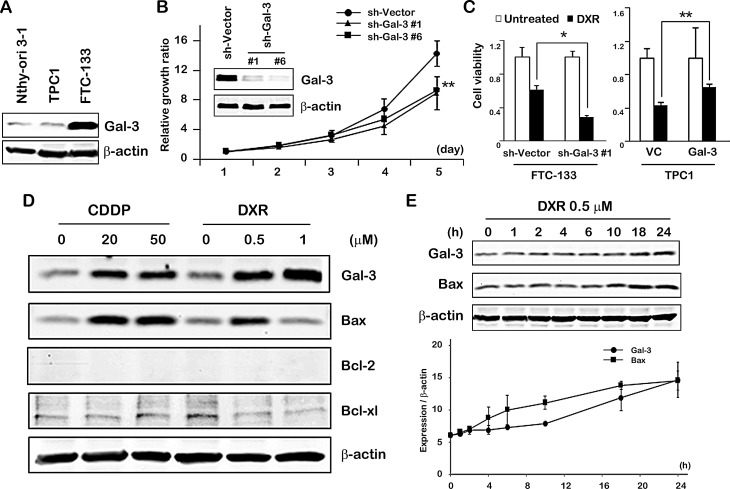
Gal-3 expression contributes to cell growth and cell death in thyroid carcinoma cells in response to DXR treatment A, Western blot analysis shows Gal-3 protein expression in Nthy-ori 3-1, TPC1 and FTC-133 cells. β-actin was used as the loading control. B, Gal-3 knockdown led to decreased cell growth of FTC-133 cells. Cell growth was analyzed by MTT assay. The value of day 1 was set as 1. **P < 0.01 vs vector. *Points* in MTT assay represent the mean of three independent experiments; *bars*, SE. C, FTC-133 and TPC1 stable cells were established as in Material and Methods. They were either untreated or treated with 1 μM DXR for 24 hours. Cell viability was determined by MTT. The value of untreated cells was set as 1. **P < 0.01, *P < 0.05. Columns represent the mean of three independent experiments; bars, SE. D, TPC1 cells were exposed to the indicated concentration of CDDP or DXR. Cell lysates were prepared and processed for western blot assay 24 hours after treatment. E, TPC1 cells were treated with 0.5 μM of DXR. Kinetic analyses of the indicated proteins were done at the times indicated. Analysis was normalized by β-actin. *Points* represent the mean of two independent experiments; *bars*, SE.

Initially, we examined the profile of Gal-3 expression in thyroid carcinoma cell lines before addressing the roles of Gal-3. We observed Gal-3 overexpression in FTC-133 cells but not in TPC1 cells compared with thyroid cells (Nthy-ori 3-1) (Figure 1A). Thus we used FTC-133 cells for stable clone with Gal-3 knockdown and TPC1 cells for overexpression of Gal-3. Consistently observed with other cancer types such as breast and prostate, we observed that Gal-3 knockdown FTC-133 cells grew slower than control cells (Figure 1B), indicative of Gal-3 role in favor of thyroid cancer growth. In addition, doxorubicin (DXR) treatment was more effective to Gal-3 knockdown FTC-133 cells than vector control. Supporting this, Gal-3 overexpressing TPC1 cells treated with DXR showed higher viability compared with vector control cells (Figure 1C). To examine the expression pattern of Gal-3 following anticancer drug treatment, TPC1 cells were exposed to cis-diammineplatinum dichloride (CDDP) and DXR, which lead to DNA double-strand breaks and induce apoptosis of cancer cells. As shown in Figure 1D, after the treatment with CDDP or DXR, we detected increased expression of Gal-3 protein as well as the pro-apoptotic protein Bax. We found that Gal-3 expression increased but Bax expression was similar to level of untreated condition when cells were treated with 1μM of DXR treatment for 24 hours. When we treated the cells with 1μM of DXR, we observed much more cell death compared with other treatments. Based on these findings, we inferred that 1μM-treated cells increased Bax expression much earlier and then the effect of cell death might compensate the difference of Bax expression (lane 4 and 6 in Figure 1D). Bcl-2, which is an anti-apoptotic protein, was not detected in TPC1 cells. Bcl-xl did not show a significant change with CDDP treatment and decreased with DXR treatment. Furthermore, we investigated the kinetics of Gal-3 and Bax after treatment with DXR (Figure 1E) and the analysis showed that Gal-3 and Bax increased gradually in response to DXR. The data that Bax is induced by DXR in TPC1 cells indicated the induction of apoptosis. Intriguingly, it is likely that anti-apoptotic Gal-3 was induced in order to protect cells in response to severe apoptotic stimuli.

### Gal-3 contributes to anti-apoptosis in intrinsic apoptotic pathway

**Fig.2:**
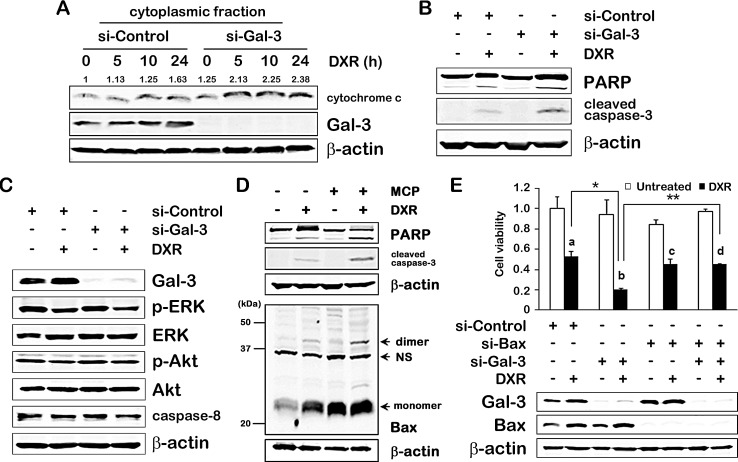
Gal-3 contributes to anti-apoptosis in intrinsic apoptotic pathway A, TPC1 cells were transfected with si-Control or si-Gal-3 for 24 hours, and then either untreated or treated with 1 μM of DXR for 24 hours. The release of cytochrome c was determined in the cytoplasmic fraction of TPC1 cells at the times indicated. The quantification of band intensity was performed using ImageJ software and the value of si-Control for 0 hour was set as 1. B and C, TPC1 cells were treated as in A. Indicated protein expressions were determined. β-actin was used as the loading control. D, TPC1 cells were pretreated with 1% of GCS-100/MCP for 3 hours, and then either left untreated or treated with 1 μM DXR for 24 hours. (Upper) Cell lysates were isolated, and PARP cleavage levels or cleaved caspase-3 expression were determined. (Lower) Bax oligomerization assay. Cells were cross-linked with 1, 6-bismaleimidohexane (BMH) and immunoblotted with polyclonal anti-Bax antibody. NS means non-specific band. E, TPC1 cells were transfected with indicated siRNA for 24 hours, and then either untreated (white columns) or treated with 0.5 μM of DXR (black columns) for 24 hours. Western blot analysis shows Gal-3 and Bax protein expression. Cell viability was analyzed by MTT assay. The value of untreated control cells was set as 1. The symbols of a, b, c and d indicate the black columns of lane 2, 4, 6 and 8 in the graph, respectively. **P < 0.01, *P < 0.05. *Columns* represent the mean of three independent experiments; *bars*, SE.

To further explore how Gal-3 is linked to apoptotic pathways, we first performed suppression studies in which DXR-induced apoptosis was expected to be enhanced following siRNA-mediated knockdown of Gal-3. We firstly analyzed whether Gal-3 was involved in intrinsic apoptotic pathway, which was characterized by permeabilization of the mitochondria and release of cytochrome c into the cytoplasm. In cells in which Gal-3 was knocked down, there was an increase in cytoplasmic cytochrome c at 5 hours after DXR treatment, while in control cells, at 10 hours (Figure 2A). Consistently, following the release of cytochrome c, activation of caspase-3 and poly (ADP-ribose) polymerase (PARP) cleavage in Gal-3 knockdown cells were increased compared with control cells (Figure 2B), indicating that DXR-induced apoptosis is enhanced by siRNA against Gal-3 and anti-apoptotic function of Gal-3 is participating in intrinsic apoptotic pathway. In thyroid cancer cells, Gal-3 was reported to induce PI3K-Akt pathway in which acts as pro-survival signaling and inhibits pro-apoptotic sensors such as BID [14]. The extrinsic apoptotic pathway is induced by death receptors such as tumor necrosis factor receptor 1 (TNFR1) and Fas/CD95. Ligands bind to these receptors, which form the death inducing signaling complex (DISC), leading to initiation of the caspase cascade through caspase-8 [4]. To examine whether Gal-3 affects extrinsic pathway or alternative apoptotic pathway, we analyzed caspase-8, p-ERK and p-Akt and did not find significant difference in expression of these factors under Gal-3 knockdown (Figure 2C), indicating the anti-apoptotic role of Gal-3 on the intrinsic apoptotic pathway. Next, to further delineate how Gal-3 plays in intrinsic pathway, we examined Bax oligomerization in response to apoptotic stimulus [15]. We used the GCS-100/modified citrus pectin (MCP) which shows inhibitory effect by targeting the CRD of Gal-3 [16, 17]. The backbone of MCP is a galacturonic acid and is an antagonist. Although the question whether MCP binds to other galectins is reasonable, as other galectins may be involved in apoptosis [18, 19], none have been linked to Bax-mediated intrinsic pathway. As a result, Bax oligomers along with PARP cleavage and activation of caspase-3 were induced in DXR treated cells, but not in untreated cells. Interestingly, treatment with GCS-100/MCP and DXR together enhanced Bax oligomerization (Figure 2D). These results indicate that endogenous Gal-3 protects DXR-induced apoptosis through CRD of Gal-3, and Gal-3 directly or indirectly inhibits Bax oligomerization. Cell viability assay showed a slower growth rate in Gal-3 knockdown cells (b) after DXR treatment than in control cells (a). However, double knockdown cells with siRNA against Bax and Gal-3 with DXR treatment (d) did not exhibit the inhibition of cell viability when compared to knockdown of Bax alone (c) (Figure 2E). It indicated that low levels of Gal-3 facilitated apoptosis induced by DXR on the intrinsic pathway, and the anti-apoptotic effect of Gal-3 was mediated by Bax.

### Gal-3 binds to Bax through CRD in response to apoptotic stimulus

**Fig.3:**
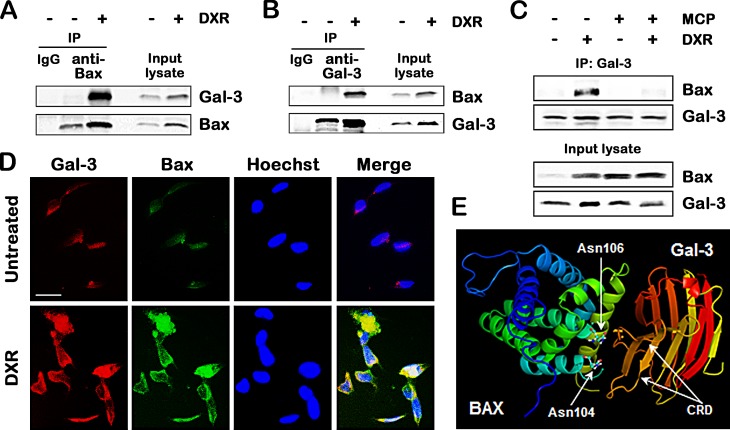
Gal-3 binds to Bax through CRD in response to apoptotic stimulus A and B, Co-immunoprecipitation assay. TPC1 cells were treated with 0.5 μM DXR for 24 hours. Cell lysates were immunoprecipitaed with IgG rabbit, polyclonal anti-Bax, or polyclonal anti-Gal-3 antibody. The immunoprecipitates and input lysates were analyzed by immunoblotting with indicated antibodies. Input lysates indicate lysates used for immunoprecipitation from TPC1 cells and were used as positive control. C, TPC1 cells were pretreated with 1% of GCS-100/MCP for 3 hours, and then either left untreated or treated with 1 μM DXR for 24 hours. Cell lysates were immunoprecipitated with polyclonal anti-Gal-3 antibody. The immunoprecipitates and input lysates were analyzed by immunoblotting with indicated antibodies. D, Co-localization of Gal-3 and Bax in TPC1 cells treated with 0.5 μM DXR for 24 hours. TPC1 cells were immunofluorescently labelled with anti-Gal-3 (red), anti-Bax (green) antibodies and Hoechst 33258 (nuclear stain, blue). Scale bar represents 50 μm. E, Prediction of the interaction of Gal-3 carbohydrate recognition domain (CRD) with Bax. The references about the structure of Gal-3 CRD and Bax were indicated in Materials and Methods. *In silico* docking was performed using Second ClusPro 2.0 server (http://cluspro.bu.edu/login.php). Asn means asparagine.

Since Gal-3 affected Bax oligomerization, we addressed the rising possibility that Gal-3 directly interacted with Bax. As shown in Figure 3A, co-immunoprecipitation assay showed that the anti-Bax-immunoprecipitates contained endogenous Gal-3 after DXR treatment. Consistent with these results, the reciprocal experiments revealed that endogenous Bax was co-immunoprecipitated with Gal-3, but not without DXR (Figure 3B). It is poorly understood that DXR treatment enhances interaction of both proteins. Because GCS-100/MCP enhanced DXR-induced apoptosis, we next validated whether the interaction of Gal-3 with Bax was mediated by CRD of Gal-3. In cells treated with GCS-100/MCP and DXR together, the interaction of Gal-3 with Bax was suppressed significantly (Figure 3C), indicating that Gal-3 binds to Bax through CRD. In addition, we performed immunofluorescence study whether Gal-3 was co-localized to Bax. Co-localization of Gal-3 and Bax was revealed in the cytoplasm of the cells treated with DXR, suggesting that they are physically proximal (Figure 3D). Furthermore, taking advantage of the data obtained from the structures of the CRD of Gal-3 and Bax, we performed *in silico* docking analysis using Second ClusPro 2.0 server to predict their physical interactions. *In silico* docking analysis suggested that Gal-3 CRD bound within the BH1 domain of Bax containing asparagine 104 and 106 of the NWGR motif (Figure 3E), whereas Bcl-2 anti-apoptotic proteins bound BH3 domain of Bax [5].

### NWGR motif of Gal-3 CRD is crucial for interaction with Bax

**Fig.4:**
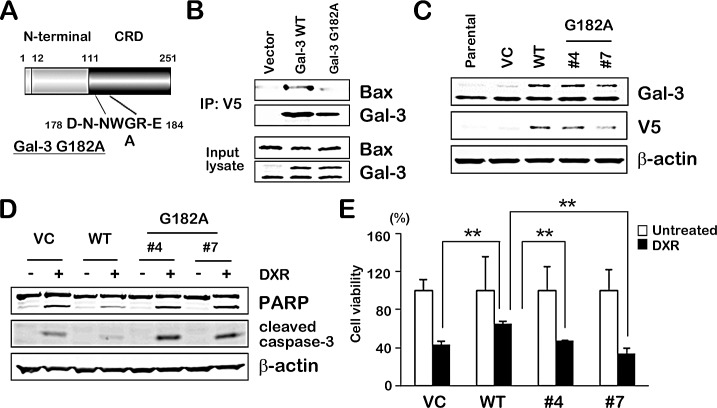
NWGR motif of Gal-3 CRD is crucial for interaction with Bax A, Gal-3 is composed of three structural domains: (a) a NH2-terminal domain of 12 amino acids; (b) a repeated collagen-like sequence rich in glycine, proline, and tyrosine; and (c) a COOH-terminal CRD. The C-terminal domain includes the NWGR motif. Wild type Gal-3 and mutant Gal-3 G182A were generated and cloned into the pcDNA6/V5 expression vector. B, Indicated plasmids were transiently transfected into 293 cells. After 48 hours, cell lysates were immunoprecipitaed with anti-V5 antibody. The immunoprecipitates and input lysates were analyzed by immunoblotting with indicated antibodies. Input lysates from 293 cells were used as positive control. C, Western blot analysis shows Gal-3 and V5 protein expression in TPC1 stable clones. Wild type Gal-3 and mutant Gal-3 G182A in pcDNA6/V5 expression vector were used for establishment of stable clones (vector control; VC, wild type Gal-3; WT and mutant Gal-3 G182A; #4 and #7). β-actin was used as the loading control. D, TPC1 stable cells were either untreated or treated with 1 μM DXR for 24 hours. PARP cleavage levels and cleaved caspase-3 expression were determined. β-actin was used as the loading control. E, TPC1 stable cells were treated as in D. Cell viability was analyzed by MTT assay. Cell viability of treated cells was represented as the percentage of untreated control cells. **P < 0.01 vs untreated cells. *Columns* represent the mean of three independent experiments; *bars*, SE.

As it suggested that the NWGR motif in the CRD of Gal-3 was pivotal to anti-apoptotic function of Gal-3 [11, 12], we determined the significance of the NWGR motif in Gal-3 for Gal-3/Bax interaction. We constructed mutant Gal-3 (glycine 182 to alanine; G182A) using site-direct mutagenesis (Figure 4A). Co-immunoprecipitation study in transiently transfected 293T cells revealed that mutant Gal-3 G182A weakly bound to Bax, suggesting the deficiency of Gal-3 functionality (Figure 4B). Furthermore, we established two stable clones transfected with Gal-3 mutant and differentially selected in TPC1 cells in order to confirm characteristics of Gal-3 mutant in apoptotic signaling pathway (Figure 4C). We examined whether mutant Gal-3 affected PARP cleavage and activation of caspase-3 in stable clones under DXR treatment. PARP cleavage and activation of caspase-3 were significantly decreased in WT cells compared with VC cells or mutant Gal-3 clones (Figure 4D), indicating that WT overexpression suppressed PARP cleavage and activation of caspase-3 but vector and mutant was similar. Consistent with a reduction of Gal-3 mutant functionality in apoptotic pathway, mutant clones demonstrated a reduced anti-apoptotic function in the cell viability test (Figure 4E). These data showed that overexpression of Gal-3 led to the attenuation of apoptosis in TPC1 cells, and an amino acid substitution in the NWGR motif of Gal-3 abrogated the attenuation of apoptosis.

### Anti-apoptotic role of Gal-3 through Bax is suppressed by Gal-3 inhibitor in cancer cells

**Fig.5:**
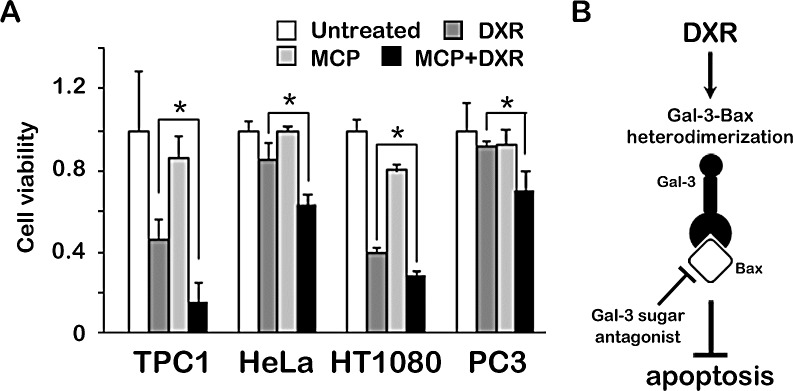
Anti-apoptotic role of Gal-3 through Bax is suppressed by Gal-3 inhibitor in cancer cells A, TPC1, HeLa, HT1080 and PC3 cells were pretreated with 1% of GCS-100/MCP for 3 hours, and then either left untreated or treated with 1 μM DXR for 24 hours. Cell viability was determined by MTT. The value of untreated cells was set as 1. *P < 0.05. *Columns* represent the mean of three independent experiments; *bars*, SE. B, Schematic representation for the regulation of apoptosis of Gal-3 by binding to Bax. Gal-3 heterodimerizes Bax, mediated by CRD of Gal-3, leading to anti-apoptotic characteristic. Gal-3/Bax interaction was suppressed by sugar antagonist of Gal-3, in which in turn cells became sensitive to apoptosis.

Finally, we wonder whether our finding of Gal-3/Bax interaction in thyroid carcinoma cells is extended to other cancer cells [20-22] and examined cell viability with Gal-3 inhibitor under apoptotic stimulus. The results showed that the treatment of Gal-3 inhibitor sensitized cells to DXR-induced cell death regardless of cancer cell types (Figure 5A). These data indicate that Gal-3 inhibitor confers cells sensitivity to DXR-induced apoptosis, suggesting that the mechanisms for anti-apoptotic role of Gal-3 through Bax can be broadly applied to human cancers.

## DISCUSSION

In thyroid cancer, the mutant genes such as BRAF and RAS constitutively activate aberrant cell signaling pathways that control apoptosis through MAPKs signaling [23]. MAPKs signaling pathway can function as a pro- or anti-apoptotic factor by switching to turn on or off the substrate proteins, including p53 and Bcl-2 family proteins [24]. Following the different etiology of thyroid carcinoma, the cancer cells commonly showed anti-apoptotic characteristics for their survival. The mechanisms for apoptotic resistance remain to be elucidated in thyroid cancer. Thus, we addressed the contribution of Gal-3 to apoptosis in thyroid carcinoma, based on that Gal-3 is clinically associated with malignant transformation of thyroid carcinoma [25, 26].

The studies presented earlier have shown that intracellular Gal-3 imparts resistance to apoptosis in breast, bladder and prostate cancer cells [27, 28], whereas recombinant Gal-3 is also exogenously added and enhances cancer progression [29]. In most of reports about the association of Gal-3 to the resistance of drug-induced apoptosis in several types of cancer cells, the anti-apoptotic function of Gal-3 is documented through promising molecular findings. These include the modification of phosphorylated Gal-3, its nuclear export to mitochondria [30-32], suppression of TNFR1-induced extrinsic apoptotic pathway [33] or the activation of alternative pathways such as PI3K pathway for pro-survival [14, 34]. Apart from these indirect evidences, we demonstrate in this study that Gal-3 plays a direct role in the modulation of intrinsic apoptosis in thyroid carcinoma cells (Figure 2).

Although it is well known that interactions of pro-apoptotic Bax/Bak and anti-apoptotic Bcl-2 family members through protein crystallography [3], It is intriguing that cytosolic Gal-3 interacts with Bax, leading to inhibition of Bax oligomerizaiton instead of role of mitochondrial membrane-spanning Bcl-2 family. Here, the question includes that Gal-3 acts as (i) a modulator in Bcl-2/Bax complex or (ii) a sole regulator of Bax independently of anti-apoptotic Bcl-2 members. For the former mechanisms, we addressed the rising possibility that Gal-3 is involved in Bcl-2/Bax heterodimer because anti-apoptotic Bcl-2 normally sequesters pro-apoptotic Bax on mitochondria. *In silico* docking analysis also suggested that Gal-3 CRD can bind to the BH1 domain of Bax (Figure 3E) instead of BH3 which mediate Bcl-2/Bax heterodimerization [35]. However, we did not obtain promising data on a triple complex (Gal-3, Bax and Bcl-2) when co-immunoprecipitation assays were performed (data not shown). Since Bcl-2 family consists of many members and expresses in cell-type specific fashion [4], further studies will be needed to define the role of Gal-3 using constructs of these Bcl-2 members.

It is supported that cell death was partially overcome by Gal-3 overexpression when cells were exposed to an apoptotic drug (Figure 4). It opens the possibility that Gal-3-overexpressing cancer cells have a survival advantage. Given that Gal-3 overexpression is clinically associated with human thyroid carcinoma and other various cancers, Gal-3 targeting could be considered in an improved therapeutic modality for cancers. Although thyroid cancer cells remain to be explored in combination with typical Bcl-2 inhibitor and Gal-3 inhibitor, targeting Gal-3 might be effective to improve the efficacy of Bcl-2 targeted therapy [36].

## MATERIAL AND METHODS

### Materials and antibodies

Cis-diammineplatinum dichloride (CDDP), doxorubicin hydrochloride (DXR) and thiazolyl blue tetrazolium bromide (MTT) were purchased from Sigma-Aldrich (St. Louis, MO).

Monoclonal rat anti-Gal-3 antibody was isolated from the supernatant of hybridoma (catalog number: TIB-166, American Type Culture Collection, Manassas, VA); customized polyclonal rabbit anti-Gal-3 antibody was created by Invitrogen (Carlsbad, CA); mouse anti-V5 was purchased from Invitrogen; polyclonal rabbit anti-Bax, mouse anti-Bcl-xl, mouse anti-p-ERK, mouse ERK1/2 and rabbit anti-p-Akt were purchased from Santa Cruz Biotechnology (Santa Cruz, CA); mouse anti-β-actin was purchased from Sigma-Aldrich; monoclonal mouse anti-Bax was purchased from BD Biosciences (San Diego, CA); mouse anti-p21, rabbit anti-Bcl2, rabbit anti-PARP (9542L), rabbit anti-cleaved caspase-3, rabbit anti-Akt and mouse anti-caspase-8 were purchased from Cell Signaling Technology (Beverly, MA); purified rabbit IgG was purchased from ZYMED (San Francisco, CA);

### Cells

Human thyroid carcinoma cells TPC1 were obtained from the University of Colorado Cancer Center Cell Bank (Denver, CO). FTC-133 was obtained from the University of California Cell Culture Core Facility (San Francisco, CA). Human thyroid cells Nthy-ori 3-1 were purchased from Sigma-Aldrich. 293 cells, HeLa cells, HT1080 cells and PC3 cells were purchased from American Type Culture Collectioun. These cell lines have been tested and authenticated by the supplier. FTC-133, Nthy-ori 3-1, 293, HeLa, HT1080 and PC3 cells were cultured in Dulbecco's modified Eagle's medium (DMEM) supplemented with 10% fetal bovine serum (FBS), and the other cells were cultured in RPMI 1640 supplemented with 10% FBS. To maintain stable clones, 200 μg/ml of G418 and 10 μg/ml of blasticidin (Invitrogen) were added to the culture media for FTC-133 and TPC1 transfectants, respectively.

### Plasmids and siRNA transfection

Oligonucleotides sequence of small hairpin (sh)-RNAs against Gal-3: 5′-GATCCCGGGAAGAAAG-ACAGTCGGTTTCAAGAGAACCGACTGT CTTTCTTCCCTTTTTTGGAAA-3′. Sh-RNA expression plasmid targeting Gal-3 and control plasmid were synthesized and annealed by Thermo Scientific (Pittsburg, PA). It was subcloned into pSilencer 3.1-H1 neo expression vector (Ambion, Austin, TX). FTC-133 stable clones were selected by 600 μg/ml of G418.

siRNA against Gal-3 and control siRNA (Santa Cruz Biotechnology) were transfected into each cells using Lipofectamine RNAiMax reagent (Invitrogen) according to the manufacturer's instruction.

Expression plasmid of Gal-3-V5 was constructed in pcDNA6/V5 harboring human full-length Gal-3 and confirmed by sequence analysis. 293 Cells were seeded at 50% confluence per well in plates overnight and transfected transiently for 48 hours using Lipofectamine LTX and Plus Reagent (Invitrogen) according to the manufacturer's instructions. TPC1 stable clones were selected by 10 μg/ml of blasticidin.

### RNA extraction and Reverse Transcription-PCR (RT-PCR)

Total RNA was extracted with Trizol reagent (Invitrogen). For the RT reaction, 2 μg of total RNA was used by using the First-Strand cDNA synthesis kit (GE Healthcare, Piscataway, NJ) according to the manufacturer's instruction. In PCR reaction, 1 μl of the resultant cDNA from the RT reaction was used as the template.

The following primers were used for RT-PCR amplification. Gal-3 was amplified with primers (forward: 5′-GCCACTGATTGTGCCTTA-3′ and reverse: 5′-AACCGACTGTCTTTCTTCC-3′). Glyceraldehyde 3-phosphate dehydrogenase (GAPDH) was amplified with primers (forward: 5′-GATGACATCAAGAAGGTGGTGA-3′ and reverse: 5′-TTCGTTGTCATACCAGGAAATG-3′). PCR products were electrophoresed on 0.5% agarose gel and stained with ethidium bromide. Density of each band was visualized using an Odyssey Infrared Imaging System. All experiments were carried out in quadruplicates and repeated twice. Statistical analysis was done using paired Student *t*-test. *P < 0.05* was regarded as significant.

### Preparation of cytoplasmic extracts

Cells were washed with phosphate buffered saline (PBS) and centrifuged at 500 *g* for 5 minutes. The supernatant was discarded and the cell pellet was used for extraction of the cytoplasmic fractions using cytoplasmic extraction reagents (Pierce Biotechnology, Rockford, IL) according to the manufacturer's instructions.

### Western blot assay

Cells were lysed in buffer (50 mM Tris-HCl pH 7.4, 1% NP-40, 0.5% Na-deoxycholate, 0.1% SDS, 150 mM NaCl, 2 mM EDTA, 50 mM NaF and 0.2 mM Na_3_VO_4_) containing protease inhibitors (Roche Applied Science, Nutley, NJ). After BCA protein assay (Pierce Biotechnology), equal amounts of proteins were separated on SDS-polyacrylamide gel electrophoresis gels and transferred to polyvinylidene fluoride membranes (Millipore, Bedford, MA). Membranes were blocked in 0.1% casein/ Tris buffered saline (TBS) for 1 hour, incubated with appropriate primary antibodies for overnight at 4°C, then incubated with secondary antibodies conjugated with IRDye 800 (Rockland Immunochemicals, Gilbertsville, PA) or Alexa Fluor 680 (Invitrogen) for 1 hour at room temperature. Membranes were washed three times with TBS including 0.1% Tween20 at 5 minutes intervals, and were visualized using an Odyssey Infrared Imaging System. Relative protein levels were quantified using the ImageJ software (National Institute Health), and normalized to β-actin. Each experiment was repeated at least, twice.

### Immunofluorescence

Cells were fixed with 4% paraformaldehyde in PBS for 15 minutes, permeabilized with 0.25% Triton X-100 for 10 minutes, blocked with 1% bovine serum albumin (BSA) in PBS for 30 minutes and incubated with indicated antibodies for overnight, then incubated with tetramethylrhodamine isothiocyanate (TRITC)-conjugated antibody and fluorescein isothiocyanate (FITC)-conjugated antibody (Sigma-Aldrich) for 1 hour in the dark. Nuclei were stained with 2.5 μg/ml Hoechst 33258 (Invitrogen) for 5 minutes. Pictures were taken using same parameters with OLYMPUS BX40 microscope (Melville, NY) and CellSens Dimension Imaging Software (Olympus).

### Co-Immunoprecipitation assay

Cells were lysed in previously described buffer containing 1% CHAPS and protease inhibitors (Roche Applied Science). After BCA protein assay (Pierce Biotechnology), cell lysates containing equal amount of proteins were incubated with appropriate antibodies overnight and 15 μl of protein G Sepharose (GE Healthcare) for 1 hour at 4°C. The beads were washed three times and boiled in 2x sample buffer. Supernatant was subjected to SDS-polyacrylamide gel electrophoresis and immunoblotted for appropriate antibodies. Each experiment was repeated at least, twice.

### MTT assay

Briefly, cells were seeded at well in 24-well plates. At the time of assay, 0.1 mg/ml MTT in basic medium was added to each well and incubated for 1 hour. After removing MTT, dimethyl sulfoxide was added and mixed vigorously. Absorbance was measured at 485nm. All experiments were carried out in quadruplicates and repeated twice. Statistical analysis was done using paired Student *t*-test. *P < 0.05* was regarded as significant.

### Preparation of GCS-100/MCP

Citrus Pectin (CP) was purchased from Sigma Chemicals. Temperature modification of CP was performed as follows: CP solution (1.3%) was autoclaved for 1 hour, cooled to room temperature, centrifuged at 10,000 *g* for 10 minutes. Collected supernatant was precipitated with 2 volumes of absolute ethanol and frozen at −20°C for 2 hours. After centrifuging at 10,000 *g* for 10 minutes again, the supernatant was discarded and pellet was saved. The pellet was suspended in acetone, filtered, and dried on Whatman filters. MCP was dissolved in de-ionized distilled water.

### In silico docking analysis

We used Second ClusPro 2.0 server (http://cluspro.bu.edu/login.php) to perform rigid-body docking of Gal-3 CRD (1A3K) with Bax (1F16) from RCSB PDB (http://www.rcsb.org/pdb/home.do).

### Detection of Bax oligomerization

For analysis of Bax oligomerization in cells, washed cell pellets were resuspended in PBS with freshly prepared 1, 6-bismaleimidohexane (Thermo Scientific) at a final concentration of 5 mM and incubated with rotation for 30 minutes at room temperature. The cells were pelleted, dissolved in buffer, incubated on ice for 5 minutes, and centrifuged at 16,000 *g* for 10 minutes. Supernatants were analyzed by immunoblotting with polyclonal anti-Bax antibodies, after BCA protein assay (Pierce Biotechnology) [37].
